# An fMRI Study of Nicotine-Deprived Smokers' Reactivity to Smoking Cues during Novel/Exciting Activity

**DOI:** 10.1371/journal.pone.0094598

**Published:** 2014-04-11

**Authors:** Xiaomeng Xu, Arthur Aron, J. Lee Westmaas, Jin Wang, Lawrence H. Sweet

**Affiliations:** 1 Idaho State University, Pocatello, Idaho, United States of America; 2 Stony Brook University, Stony Brook, New York, United States of America; 3 American Cancer Society, Atlanta, Georgia, United States of America; 4 Chinese Academy of Sciences, Beijing, China; 5 University of Georgia, Athens, Georgia, United States of America, and Warren Alpert Medical School of Brown University, Providence, Rhode Island, United States of America; University of Pennsylvania, United States of America

## Abstract

Engaging in novel/exciting (“self-expanding”) activities activates the mesolimbic dopamine pathway, a brain reward pathway also associated with the rewarding effects of nicotine. This suggests that self-expanding activities can potentially substitute for the reward from nicotine. We tested this model among nicotine-deprived smokers who, during fMRI scanning, played a series of two-player cooperative games with a relationship partner. Games were randomized in a 2 (self-expanding vs. not) x 2 (cigarette cue present vs. absent) design. Self-expansion conditions yielded significantly greater activation in a reward region (caudate) than did non-self-expansion conditions. Moreover, when exposed to smoking cues during the self-expanding versus the non-self-expanding cooperative games, smokers showed less activation in a cigarette cue-reactivity region, *a priori* defined [temporo-parietal junction (TPJ)] from a recent meta-analysis of cue-reactivity. In smoking cue conditions, increases in excitement associated with the self-expanding condition (versus the non-self-expanding condition) were also negatively correlated with TPJ activation. These results support the idea that a self-expanding activity promoting reward activation attenuates cigarette cue-reactivity among nicotine-deprived smokers. Future research could focus on the parameters of self-expanding activities that produce this effect, as well as test the utility of self-expansion in clinical interventions for smoking cessation.

## Introduction

Research from several disciplines support the idea that reward from one domain can replace or substitute for reward in another. One example is clinical accounts of drugs or behaviors with similar hedonics substituting for one another [1]–[3]. This phenomenon also occurs in response to public policy, such as when adolescent rates of marijuana smoking increase following more stringent regulations on underage drinking [4]–[5], and experimentally with food and drug rewards in animal models. For example, Wellman and colleagues [6] found that rats maintained on a high-fat diet (compared to rats maintained on a standard pellet diet) exhibited diminished acquisition of self-administered cocaine.

The mechanism for this reward substitution/replacement effect involves the neurotransmitter dopamine, which is strongly linked to the reinforcing effects of drugs [7]–[8]. Dopamine and the mesolimbic dopaminergic system of the brain are also involved in the rewarding and motivating aspects of social relationships. Social relationship reward has been shown to substitute for food among young adults [9], and social bonding has been shown to decrease amphetamine reward (mediated through dopamine) among prairie voles, a monogamous mammalian species [10].

Social relationships may be rewarding because they are often a form of “self-expansion.” The self-expansion model states that people are motivated to expand their identity and increase their physical, informational, and social resources [11]. Self-expansion is rooted in approach motivation [12] and is characterized by novelty, excitement, and interest or challenge, such that those who engage in these self-expanding activities experience a sense of growth in their self-concept [11]. Relationship self-expansion (e.g., via forming romantic relationships) activates the mesolimbic dopamine pathway [13]–[21]. Xu et al. [22] conducted a functional magnetic resonance imaging (fMRI) study of nicotine-deprived smokers who were in the early stage of a romantic relationship (and reported intense love for their partner as assessed by the Passionate Love Scale; PLS) [23]. Viewing images of their partner (as opposed to a familiar acquaintance) while exposed to cigarette cues elicited significantly less activation in smoking cue-reactivity brain regions.

Self-expansion also occurs outside of the social context via novelty, excitement and interest/challenge in daily life [24]–[26], and possibly other contexts as well. For example, research on self-expansion as an aid in smoking abstinence and cessation [26] found that smokers who successfully quit had experienced significantly more self-expanding events in their lives prior to their quit attempt (e.g., learning a new skill, taking an exciting course) compared with smokers who tried to quit but ultimately failed. Even among smokers who attempted to quit but failed, self-expansion appeared to be beneficial, as there was a significant positive correlation between the number of self-expanding events prior to the quit attempt and how long smokers were able to abstain.

The current research sought to build on findings by Xu et al. [22], [26] on self-expansion and smoking. It is the first fMRI study to look at self-expansion and cigarette-cue reactivity outside of the context of early-stage intense passionate love. It is also the first to utilize an experimental paradigm involving an active response task instead of having participants passively view images of their romantic partner.

In the current study, couples in long-term relationships played cooperative games together that were either self-expanding (particularly exciting, novel, and challenging) or not, and in which a cigarette cue did or did not appear. We predicted that the self-expansion reward of participating in a socially-shared novel, challenging, and exciting activity would undermine nicotine craving and result in less activation in cigarette-cue related brain areas such as the ventral striatum, temporo-parietal junction (TPJ), anterior cingulate cortex (ACC), and amygdala [27].

## Materials and Methods

### Ethics statement

This research was approved by the IRB committee of Stony Brook University and all participants provided written informed consent.

### Participants

Participants were 20 couples from the Long Island (NY) community who were in long term relationships (at least 2 years). Each couple had at least one member who currently smoked at least eight cigarettes per day and was not attempting to quit (10 couples had both members as smokers). The smoker in the couple was the one who was scanned (if both members of the couple were smokers who met inclusion criteria and scanner screening requirements, the couple decided who entered the scanner). Participants ranged in age from 19–42 years (*M* = 24.10, *SD* = 6.17). Overall, couples' relationship length ranged from 2 years to 24 years (*M* = 3.89, *SD* = 4.84). Smokers who entered the scanner (12 men, 8 women) smoked an average of 13.25 cigarettes per day (*SD* = 8.59). Smokers began smoking on average at the age of 15.85 (*SD* = 3.31) and had been smoking on average 7.97 years (*SD* = 7.59). Four of the 20 participants scanned preferred their left hand.

Only one member of each couple was scanned, significant others who were not scanned filled out questionnaires and played the co-operative games with their partners on a computer outside the scanner. In accordance with ethical standards, this study was approved by an Institutional Review Board and all participants (and their significant others) completed written informed consent.

### General procedure

After a telephone screening, the 20 couples visited the laboratory twice. Scanner participants smoked ad libitum prior to the initial session, during which they completed demographic questionnaires. Inclusion criteria for scanner participants included that it was safe them to enter scanner (e.g. no embedded metals, no history of claustrophobia etc.), and a willingness to abstain from smoking overnight. Baseline (ad libitum) breath carbon monoxide (CO) for the scanner participant was assessed on a 1 (non-smoker) to 7 (very heavy smoker) scale using the Bedfont Smokerlyzer (Bedfont Scientific Limited; Rochester, Kent, UK). Scanner participants were then asked to abstain from nicotine products for at least 8 hours prior to coming in with their partner for the second session.

During the second session, scanner participants confirmed that they had abstained from nicotine for 8 hours and had their CO levels measured with the Bedfont Smokerlyzer. While CO parts per million data was not obtained, expired CO levels (via the 1–7 scale) were used in both sessions to verify smoker and non-smoker status, to determine whether or not participants abstained from smoking as instructed, and to improve compliance. Both members of the couple then completed pre-scan questionnaires. The Inclusion of Others in the Self Scale (IOS) [28] was used to assess closeness. The IOS comprises 7 pairs of circles representing the self and the partner, and participants indicated which pair of circles best fit their relationship. The scale was scored from 0 (circles did not overlap at all) to 6 (circles overlapped almost completely). Couples also rated on a 1 (“not at all”) to 7 (“completely”) scale how happy they were generally, how happy they were in their relationship, and how committed they felt towards their partner. Prior to scanning, couples played a practice round of the two-player game to get acquainted with the set-up. After scanning, couples repeated the pre-scan questionnaires, were debriefed, thanked, and compensated for their time ($60 to each member of the couple).

### Scanning stimuli and procedure

We utilized a 3 tesla Siemens Magnetom TIM Trio scanner, and Blood Oxygen Level-Dependent (BOLD) responses were recorded. We acquired functional images via T2* Gradient-Echo EPI scans: 30 ms TE, 2500 ms TR, 80° flip angle, 220 mm FOV, 4 mm slice thickness, 64×64 matrix, 34 slices. Voxel size was 3.4×3.4×4.0 mm. High resolution whole-brain T1 images were also acquired for anatomical reference.

Scanner participants viewed stimuli via an angled mirror mounted on the RF coil and responded with their dominant hand using a four-button response box. Partners used a desktop computer with a cloned screen outside the scanner.

All participants viewed 24 blocks of stimuli split into four conditions (six blocks each) presented in random order. The four conditions were (1) self-expanding with cigarette cue (SECig), (2) self-expanding without cigarette cue (SEnoCig), (3) non-self-expanding with cigarette cue (NSECig), and (4) non-self-expanding without cigarette cue (NSEnoCig). Self-expanding (SE) conditions were programmed to be more exciting, novel, and challenging, with the game requiring more frequent and quicker responses compared with non-self-expanding (NSE) conditions. Additionally, images used to gain points in the game were novel in SE conditions, whereas in NSE conditions they consisted of images all participants had seen during the practice session. The cigarette cue image used in all conditions was that of a lit cigarette. The practice round did not contain cigarette cues and trials were 2 seconds in length.

SECig and SEnoCig conditions each contained 15 trials (1.5 seconds per trial) per block and a total of 6 blocks. NSECig and NSEnoCig conditions each contained 9 trials (2.5 seconds per trial) per block and a total of 6 blocks. Thus all conditions each totaled 135 seconds (2.25 minutes) per block.

**Figure 1:**
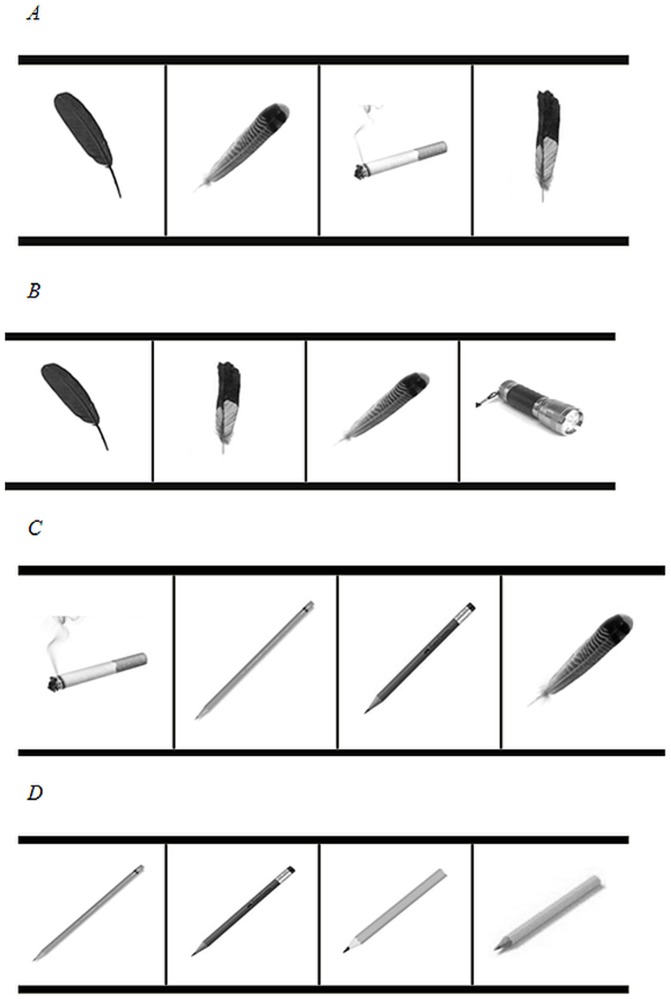
A-D: Examples of game stimuli for each condition.

Each block consisted of a series of trials where 4 black-and-white images simultaneously appeared horizontally on the screen (see Figure 1A-1D for example stimuli for each condition). Images were everyday objects (e.g., toothbrush, pencil). The goal of the two-player game was for each member of the couple to “mark” when a feather (the target) was presented by pressing buttons on the button-box (or keyboard) that corresponded to the positions of the feather. The game was cooperative such that players each got 1 point for every feather image's position they correctly marked (correct and incorrect selections were visible to both members of the couple via check marks and Xs, in each player's color, over each selected image). If both members of the couple were correct, 2 points were added to their team score (which appeared at the top of the screen throughout the game). The team score received a 1-point deduction for every incorrect selection of a non-feather image by either player.

For SECig and NSECig conditions, an image of a cigarette appeared in at least half of the trials for those blocks. In addition, self-expanding conditions (SECig and SEnoCig) contained anywhere from 0–4 target images per trial whereas non-self-expanding conditions (NSECig and NSEnoCig) contained at most 1 target image in any trial.

Following each game block, participants responded to five questions on a 1 (“not at all”) to 4 (“extremely”) scale: (a) “How much are you craving cigarettes RIGHT NOW?”; (b) “How pleasant was the game you JUST PLAYED?”; (c) “How exciting was the game you JUST PLAYED?”; (d) “How positive do you feel RIGHT NOW?”; and (e) “How negative do you feel RIGHT NOW?” These ratings were followed by a 5 second rest and the message “The next game will begin in a few seconds…”.

The protocol comprised 24 blocks of the game (six of each condition) totaling 540 seconds (9 minutes). Including the 24 rests (totaling 2 minutes), the entire protocol took 11 minutes in addition to the time participants spent responding to between-block questions.

### Analyses of fMRI Data

We used Analysis of Functional NeuroImages software (AFNI) [29] for data processing and analyses and SPSS 18.0 for follow-up analyses. Four of the 20 scanner participants exhibited excessive motion (≥4 mm) and were dropped from further fMRI analyses. Thus, results are presented for the remaining 16 participants. The 4 participants who exhibited excessive motion were significantly older and had longer smoking histories. It may be that withdrawal was more intense for these participants and thus they had more difficulty remaining still in the scanner. The 16 participants (6 women, 10 men) who did not exhibit excessive motion in the scanner ranged in age from 19–30 years (*M* = 22.44, *SD* = 3.33) and had been with their romantic partner for at least 2 years with an average of 2.91 years (*SD* = 1.11). Eight of the 16 were part of a couple in which both members smoked. These 16 scanner participants smoked an average of 11.56 cigarettes per day (*SD* = 6.01). Smokers began smoking on average at 16.81 years of age (*SD* = 3.33) and had been smoking on average 5.09 years (*SD* = 3.36). Four participants preferred their left hand.

Functional datasets were aligned to the T1 anatomical dataset, volume-registered (motion-corrected), and normalized into Talairach space [30]. The data were then spatially smoothed using a 6 mm full width at half maximum (FWHM) Gaussian filter, excluding non-brain voxels. First-level analyses of individual brain responses were characterized using general linear modeling (GLM) with regressors representing the temporal course of each condition (SECig, SEnoCig, NSECig, NSEnoCig) convolved with a gamma function. We applied eight nuisance regressors to account for motion (X, Y, Z, roll, pitch, yaw) and stimuli of no interest between blocks (rest and ratings); the GLM also incorporated linear and quadratic trends in the data. Individual *t*-statistics were generated for each condition per voxel and were used in group-level region of interest (ROI) analyses [31].

#### *A priori* anatomically defined ROI analyses

*A priori* regions identified from a meta-analysis of cigarette-cue reactivity brain areas consisted of bilateral ventral striatum, left temporo-parietal junction (TPJ; Broadmann's area 39), anterior cingulate cortex (ACC), and left amygdala [27]. For the ventral striatum, we created ROIs of 5 mm spheres centered around Kühn and Gallinat's [27] coordinates (−6, 4, −5 and 5, 5, −6). For TPJ, ACC, and amgydala, we used bilateral anatomically defined regions from the AFNI database [29]. We also used the Desikan Atlas to anatomically define the caudate (bilateral), an *a priori* reward-related region that is consistently activated during social self-expansion [14], and a region where we expected to find activation in response to our self-expansion manipulation.

#### Hypothesis testing

Comparisons for *a priori* ROI analyses were between mean parameter estimates (beta coefficients) from each region for our condition contrasts.

### Results of behavioral data

Smokerlyzer scores decreased significantly from the initial session (*M* = 3.31, *SD* = 1.25) to the scan session (*M* = 1.25, *SD* = 0.58); *t*-statistic (degrees of freedom): *t*(15) = 7.34, *p*<.001. Immediately prior to scanning smokers' general happiness averaged 4.94 (*SD* = 1.48). After the scanning (during which they played the 2-player games with their partner), general happiness increased significantly, *t*(15) = −2.15, *p* = .049, to an average of 5.75 (*SD* = 0.86). Smokers' relationship happiness also increased significantly from pre-scan (*M* = 5.94, *SD* = 1.00) to post-scan: *M* = 6.19, *SD* = 0.83; *t*(15) = −2.24, *p* = .041. Smokers' closeness to their partner (via the IOS) increased from an average of 3.87 (*SD* = 1.15) pre-scan to an average of 4.25 (*SD* = 1.29) post-scan, although this was not statistically significant, *t*(15) = -1.38, *p* = .188. Finally, smokers' commitment to their partner increased from an average of 6.63 (*SD* = 0.72) pre-scan to an average of 6.75 (*SD* = 0.45) post-scan, although this was not statistically significant *t*(15) = −1.46, *p* = .164.

#### Manipulation check: self-report during scanning

There was a significant overall main effect of game condition on excitement, *F*(1,380) = 4.41, *p* = .005. Follow-up *t*-tests indicated that game excitement ratings for the SECig condition were significantly higher (*M* = 2.32, *SD* = 0.91) than for the NSECig condition [*M* = 2.04, *SD* = 0.88; *t*(380) = 2.09, *p* = .038], and significantly higher for the SEnoCig condition (*M* = 2.43, *SD* = 0.98) than for the NSEnoCig condition [*M* = 2.03, *SD* = 0.96; *t*(380) = 2.94, *p* = .004]. There were no significant main effects of game condition on pleasantness, positive mood, or negative mood. This was in line with the goals of game development; we were aiming to have the self-expanding and non-self-expanding games differ on excitement alone (while exhibiting no differences on pleasantness or affect) as excitement is a main component of self-expansion.

Due to technical issues during scanning, in-scanner self-reported craving ratings were not recorded for three of our 16 scanner participants (these participants still completed pre and post scan questionnaires). Based on the remaining in-scanner data (from 13 scans), self-reported ratings of craving did not differ across conditions. As we asked participants to rate their craving every 30 seconds, and on a scale that offered only 4 options, this rating system may not have been sensitive enough to pick up on variances in subjective craving. This paper therefore focuses on objective brain responses to cues.

## Results

### Results of fMRI Data

#### *A priori* anatomically defined ROIs

We found, as expected, a significant main effect of self-expansion on activation of the caudate, *t*(15) = 4.30, *p* = .001. We also found a significant main effect of cigarette cue conditions in the TPJ, *t*(15) = 3.81, *p* = .002. The hypothesized main effect of cigarette cue condition in the amygdala did not reach significance, *t*(15) = 1.93, *p* = .072. Cigarette-cue conditions did not elicit a significant response in the ACC or ventral striatum ROIs compared to neutral cue conditions, therefore we used only TPJ and amygdala for hypothesis testing. In line with our hypothesis, there were significant interactions such that during the self-expanding games, there was less activation in the TPJ, *t*(15) = −2.36, *p* = .032, compared to during the non-self-expanding games. Results were similar in the amygdala but did not reach significance *t*(15) = −1.76, *p* = .099.

#### Exploratory analysis

**Figure 2:**
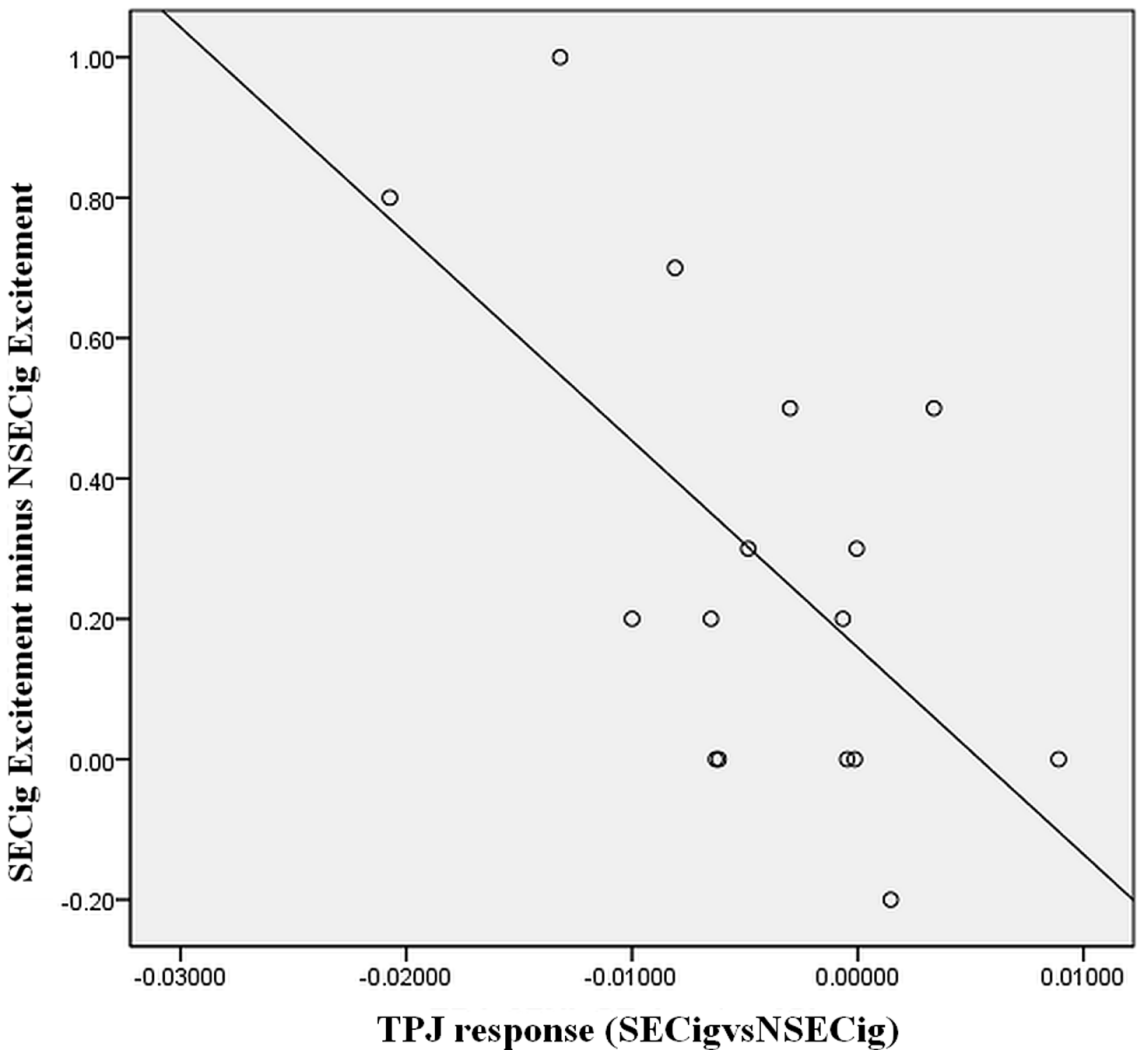
Composite self-reported game excitement (Avg SECig excitement – Avg NSECig excitement) and TPJ activation in the hypothesized contrast (SECig vs NSECig).

We also wanted to explore whether ratings of excitement associated with self-expansion were related to brain cue reactivity. We first calculated a composite self-expansion excitement score for each scanner participant by taking the average self-reported excitement score for the SECig condition and subtracting the average self-reported excitement score for the NSECig condition. This composite score (average SECig excitement – average NSECig excitement) can be considered the level of excitement during the self-expansion game (when cigarette cue was present) above and beyond the excitement of the non-self-expansion game (when cigarette cue was present). This composite excitement score was significantly negatively associated with cue-reactivity (SECig vs NSECig activation) in the TPJ, *p* = .012 (see Figure 2).

Finally, we conducted a series of exploratory *t*-tests to investigate whether results would differ by the sex of the scanned participant or by the smoking status of the non-scanned partner. We found no significant differences by sex or smoking composition of the couple, however our power was low for these exploratory analyses and thus it is possible that differences exist. Future studies with larger samples should specifically aim to investigate these factors.

## Discussion

The results of this study provide support for the reward replacement/substitution model by demonstrating that self-expansion (novel, exciting, and interesting/challenging activity with a partner) can act as a reward to attenuate brain reactivity to cigarette cues.

Specifically, we developed a cooperative 2-player game and varied its level of self-expansion by manipulating its novelty (whether the visual stimuli presented were novel or not) as well as its challenging qualities (how many targets there were and how long participants had to respond), with the expectation that these two manipulations would influence its excitement. Indeed, participants rated the self-expansion games significantly more exciting than they did the non- self-expansion games, and the self-expansion games elicited significantly greater activation in the caudate, a reward-related region associated with self-expansion in the context of intense romantic love and in the context of rewarding interactive video games [32]. The non-self-expanding games were still rated as being equally pleasant and did not elicit significantly less positive mood or significantly more negative mood compared with the self-expanding games. This indicates that self-expansion effects were due to novelty, challenge, and excitement rather than simply positive affect.

Results also indicated that the protocol was associated with significant increases in reported general happiness and relationship happiness. Closeness and commitment to the romantic partner also increased from pre-to-post scan (although neither of these increases was statistically significant). This pattern of improvements corroborates that overall, the two-player game was self-expanding; engaging in self-expanding activities with a partner has been shown to lead to such increases [11], [33]–[35]. These improvements were evident after a simple 15–20 minute protocol that interspersed self-expansion conditions with non-self-expansion conditions, even though participants were being exposed to cigarette cues during some of these conditions.

We had hypothesized a replacement effect such that we expected more activation in cigarette cue-reactivity regions when cigarette cues were present during the non-self-expanding game compared to when cigarette cues were present during the self-expanding game. We found support for our hypothesis in the TPJ, an *a priori* anatomically defined ROI. In addition, we found that ratings of excitement during self-expansion games (above and beyond the excitement during non-self-expansion games) was significantly correlated with the attenuation effect in the TPJ, suggesting a potential dose-response effect.

These results support the proposition that engaging in self-expanding activities with a long-term partner is rewarding and can attenuate cigarette cue-reactivity among nicotine-deprived smokers. While the cooperative two-player game yielded self-reported relationship improvements and may have contributed to overall excitement, another paradigm involving other social contacts (e.g. a friend, a stranger) or at the individual level (e.g. a one-player game) would theoretically be just as effective as long as the components of self-expansion (e.g. novelty, excitement, challenge) were present. Future research should explicitly test whether the cue-reactivity attenuation effect would be present in these other contexts.

One limitation of this study is the lack of eye-tracking or other methodology to measure distraction from the cigarette cue. While it is likely that any task that is exciting will offer some distraction, it would be useful for future research to explicitly examine the amount of variance that is accounted for by distraction. However, as past research has shown that it is the reward of self-expansion (rather than distraction) that accounts for pain mitigation effects [36], it is unlikely that distraction accounts for the main effect in our study. In addition, our findings that the protocol produced improvements on relationship factors are in line with predictions from the self-expansion model but not from a model of simple distraction. This study also had a modest sample size and thus we cannot make conclusions about how results might differ based on individual or couple variables. Future research with larger samples is needed to specifically investigate the influence of demographics, relationship length, and smoking variables on self-expansion and cigarette cue attenuation.

Finally, future research could examine, among smokers attempting to quit, the extent to which attenuation of brain cue-reactivity is associated with behavioral responses such as subsequent abstinence or time to next cigarette. Positive effects found would justify developing an analogue of the game we used for deployment on smartphones, which are increasingly being used as tools for behavior change including smoking cessation [37]. Together with pharmacotherapies and other cessation strategies, such games may be a useful addition to the armamentarium of strategies to help smokers quit, a substantial number of whom do so without clinical assistance [38].
